# Challenges in Process Dissociation Measures for Moral Cognition

**DOI:** 10.3389/fpsyg.2020.559934

**Published:** 2020-11-27

**Authors:** Anton Kunnari, Jukka R. I. Sundvall, Michael Laakasuo

**Affiliations:** ^1^Department of Psychology and Logopedics, University of Helsinki, Helsinki, Finland; ^2^Department of Digital Humanities, Cognitive Science, University of Helsinki, Helsinki, Finland

**Keywords:** process dissociation, utilitarianism, deontology, measurement, psychometrics, simulation studies, validity

## Abstract

The process dissociation procedure (PDP) for moral cognition was created to separately measure two dispositions of moral judgment based on the dual-process theory of moral reasoning: deontological and utilitarian inclinations. In this paper we raise some concerns from a psychometrics perspective regarding the structure, reliability, and validity of the moral PDP as a measure of individual differences. Using two simulation studies as well as a real sample of *N* = 1,010, we investigate the psychometric properties of the moral PDP. We present novel evidence showing that (1) some correlations between PDP parameters are mathematical artifacts, and as such cannot be taken as evidence in support of a theory, (2) there are severe response inconsistencies within dilemma batteries, and (3) reliability estimates for these scores seem to be far below the accepted standards. We discuss some potential theoretical and content-related reasons for these statistical issues and their implications. We conclude that in their current form, PDP measures of utilitarian and deontological tendencies are sub-optimal for assessing individual differences.

## Introduction

Valid and accurate measurement is one of the cornerstones of scientific inquiry. The psychological sciences have had concerns about how measurement instruments are created (Hedge et al., 2018), evaluated (Flake et al., 2017), and used (Flake and Fried, 2019). This has resulted in questioning the validity of both custom-made scales (see Flake and Fried, 2019) and more established instruments (Hussey and Hughes, 2020), as well as having likely contributed to the ongoing replication crisis (Loken and Gelman, 2017).

In the present paper we raise some potential pitfalls in a measure of individual differences in utilitarian and deontological moral inclinations, the moral process dissociation procedure (PDP) developed by Conway and Gawronski (2013); from here on referenced as C&G. We describe both this measure and our concerns in length below, but the main concerns are:

1. Issues with content: heterogeneity between moral dilemmas not reflected in how scores are calculated.
2. Insufficient justification for scale structure: lack of psychometric work to examine if items function well together and whether they should be combined.
3. Insufficient reliability: lack of reliability reporting and low internal consistencies observed in the current work.
4. Artefactual validity evidence: some correlations between parameters arise solely due to mathematical formulae used to compute the moral PDP scores.

First, we will cover some background on moral psychology of utilitarianism and deontology in general: the use of moral dilemmas to measure utilitarian/deontological preferences, and the dual-process model of moral judgment. We will briefly cover the consequent methodological discussion that resulted in the development of the moral PDP, and the caveats with this model.

### Measuring Utilitarian and Deontological Dispositions

Moral psychology has put great focus on deontological and utilitarian preferences. In brief, deontological thinking sees some acts as forbidden (e.g., the intentional killing of another person) because of their inherent immorality, regardless of potential benefits. In contrast, utilitarian thinking perceives ethicality of an action based on its consequences. These moral preferences are often measured with so-called sacrificial moral dilemmas (see Christensen and Gomila, 2012, for a review). These dilemmas are typically variations of a central theme, present in the classical trolley dilemma, where the moral agent needs to personally cause the death (or other injury) of at least one person if they wish to save a larger group of people from death (or other injury).

Research using these dilemmas was the basis for the dual-process model of moral judgment (see Greene, 2007) which posited that utilitarian and deontological responses were driven by two separate cognitive processes, one fast, automatic and emotion-based (implicit) and one slow and deliberate (explicit). According to the theory, a prospect of sacrificing a person for the greater good leads to a negative emotional response that drives moral disapproval (i.e., a deontological judgment of the proposed harm), and utilitarian reasoning is possible after overriding this response.

However, C&G argued that the traditional framing of the moral dilemmas was confounded, because they did not allow for the differentiation of the contributions of two processes. Scores from these dilemmas represented a bipolar continuum where deontological and utilitarian responding were the opposite endpoints. That is, the utilitarian choice always conflicted with the deontological choice: it was not possible to know whether, e.g., a high “utilitarian” score reflected strong utilitarian tendencies, weak deontological tendencies, or both.

To address this problem C&G created a process dissociation model^1^ to measure separately the strength of utilitarian and deontological inclinations. C&G based their model on the one originally developed by Jacoby (1991) in order to clarify the relative contributions of automatic and deliberate memory processes. The stated purpose in C&Gs own words was “to provide a compelling test of the dominant dual-process account of moral judgment” (p. 220). They also stated that calculating separate parameters for utilitarian and deontological inclinations for each individual in a sample allows “researchers to use [the parameters] as measurement scores in experimental or *individual differences* designs” (p. 220, italics ours). Although the latter is our main concern here, we will also examine the claim about testing the dual-process theory.

The PDP for moral cognition computes two parameters to represent the strength of an individuals’ utilitarian (U) and deontological (D) inclinations. This is done by using two dilemma types: congruent (C), where both utilitarianism and deontology should point towards an action being impermissible; and incongruent (IC), where utilitarianism should permit the act but deontology should not. Responses to C and IC dilemmas are used to compute *U* and *D* scores according to formulae presented in Simulation study 1.

The model was later refined to include a parameter for action preference in CNI model (consequences, norms, inaction; Gawronski et al., 2016, 2017). Despite this more recent approach, studies continue to use the PDP scoring method of calculating individual utilitarianism and deontology scores for participants (e.g., Białek et al., 2019; Mata, 2019; Bostyn et al., 2020).

PD based models have been thought to clarify whether it is processes underlying deontological or utilitarian reasoning that are related to other individual differences, or affected by manipulations such as cognitive load. The effect of cognitive load as well as sex differences found using the traditional sacrificial dilemmas (Greene et al., 2008; Fumagalli et al., 2010), have been verified using the PDP or similar models (Friesdorf et al., 2015; Gawronski et al., 2017). Using the PDP, experimenters have found that utilitarian but not deontological inclinations are related to other cognitive measures such as the cognitive reflection test (Patil et al., 2020). Thus, the PDP seems to replicate results produced by more traditional measurements, but it allows for more specific inferences about where, specifically, individual differences manifest, and what kinds of processes are affected by experimental manipulations. The moral PDP has been quite popular: at the time of writing, C&G’s 2013 paper alone has been cited over 400 times, and at least 30 studies have used the measure.

There have been some concerns about the appropriateness of the sacrificial dilemma method, including the moral PDP, as measures of utilitarianism and deontology. Everett and Kahane (2020) argue that the kind of “utilitarianism” measured both by the traditional approach and by the PDP dilemmas is, in the end, not true utilitarianism but only utilitarianism with some qualifications. For example, the *U* parameter as measured by the moral PDP is not positively correlated with moral views stating an obligation to maximize good. While we think this is an important discussion, our focus is the more proximal psychometric question: even if the moral PDP only measures “utilitarianism with some qualifications”, does it measure it well?

We have found few psychometric examinations on the structure of the moral PDP. Baron and Goodwin (2020) have recently shown that participants often interpret norms and consequences in CNI dilemmas (similar in structure to moral PDP dilemmas) in a way not intended by the experimenters. Due to this, they suggested that correlations between these measures and external variables may stem from systematic variation in *understanding the dilemmas as intended* rather than any meaningful differences in moral thinking. They also noted that some effects may be driven by only one specific dilemma in a dilemma set, which raises concerns of reliability. We have similar theoretical concerns about the PDP dilemmas and will cover this in more detail in subsequent sections.

The moral PDP is claimed to function as a measurement for individual differences, and as such it must “pass” the same psychometric examinations as other scales. These include justification for combining items (here dilemmas or dilemma pairs) into scales and examining their measurement accuracy. Whereas the original PDP was used in an experimental manner, the moral PDP uses it to measure individual differences as well. This type of approach has recently gained attention for causing reliability issues^2^ (Hedge et al., 2018). C&G explicitly consider the moral PDP applicable for both purposes. We want to highlight the position we are taking here: we are not attempting to challenge the usefulness of PD models in general, nor the majority of empirical findings they have produced. We specifically examine the properties of the moral PDP as an individual difference instrument. Aggregating responses across participants can cancel out inaccuracies at the individual level, and provide more accurate estimates of process magnitudes at the group level (Calanchini et al., 2018).

Here, we raise psychometric concerns about using the PDP to quantify individual differences in utilitarian and deontological tendencies. We raise these concerns and structure the rest of the introduction in the following order: (1) content issues, (2) justification for scale structure, (3) reliability of measures, and (4) validity evidence. Thereafter, we present two simulation studies as well as an empirical study to examine the validity evidence and psychometric properties for the moral PDP as an indicator of individual differences.

### Concern 1: Content Considerations

The core assumptions of the PDP have been questioned and criticized in the context of memory research from early on (see, e.g., Graf and Komatsu, 1994; Curran and Hintzman, 1995; Russo et al., 1998). Critics have pointed to empirical results contradicting some of the core statistical assumptions in the original PDP. However, we have theoretical reservations about applying the PDP to individual differences in utilitarian moral judgment, even if the main premises of the PDP were sound in general.

Baron and Goodwin (2020) have critiqued the more refined version of the moral PDP model, i.e., the CNI model. They argued that different dilemma types in PDP/CNI models permit extensive interpretation by participants, and showed that in some dilemmas there were major disagreements between the participants and the experimenters regarding what the norms and the consequences actually are in individual dilemmas. That is, while an experimenter may interpret a specific response to a dilemma (e.g., not accepting a harm) to be an exemplar of a certain type of thinking (e.g., following a deontological norm regardless of a utilitarian motive), the responder may not agree (e.g., they have an argument for why they are in fact being utilitarian).

Potential issues in interpreting the dilemmas tie into an issue with the uniformity of stimuli. For the moral PDP, the incongruent dilemmas range from whether to avoid hitting an old lady or a young woman and her child with one’s car when it’s too late to brake, to whether to kill a young Adolf Hitler in order to prevent the Second World War (Conway and Gawronski, 2013, Appendix A, pp. 231–233). It seems likely that the magnitude of the potential utilitarian benefit (or the strength of the norm against a specific harm) would affect people’s responses, but the dilemmas are given equal weight in PDP formulae (see section“Simulation 1”). Something similar applies to the congruent dilemmas as well: although these are intended as situations where the consequences are never good enough to justify the action, there are differences in the kinds of harms and consequences. In other words, there is a hidden “ladder” within the dilemmas, where different dilemmas may test for different levels of utilitarianism or deontology (some more and some less). This is not bad in and of itself, but it is not acknowledged in the calculations (i.e., weights given to the individual dilemmas in how much they measure an inclination), and as such, is not part of the reasoning in the PDP.

The heterogeneity of stimuli ties into a larger question of whether the PDP is appropriate to dissociate processes in moral reasoning. While the PDP has been used as a content-neutral procedure for separating the contributions of two processes behind many different tasks, note that the PDP started as a way of separating contributions of (automatic and conscious) processes in *memory*, specifically word recollection. It seems perfectly reasonable to measure a person’s success in a memory task as the number of items recalled from a list. However, it is trickier to measure the *strength* of any moral inclination as the number of specific answers to a series of similar questions about harm. That is, a better performance in memory is characterized by more things remembered, so it makes sense to measure memory performance in this way. A “better performance” in, e.g., utilitarian thinking is characterized by greater acceptance towards utilitarian sacrifice, which does not translate equally well to be measured simply as a number of certain responses unless the heterogeneity in items is accounted for in the calculations.

We understand that a total uniformity of stimuli in the moral PDP would be counterproductive - it would amount to asking the same question ten times. Thus, it makes sense that the levels of utilitarian motive and/or harms vary between the dilemmas, but we feel that this may simply be a concession that becomes necessary because of the structure of the PDP. Since there is no clear ranking of the dilemmas (as in an Item Response Theory approach), or a *priori* knowledge of how strong the deontological norms for each dilemma are, it is unclear how much utilitarian or deontological inclinations actually affect a specific response. In our empirical data, we find not only “hard” and “easy” items in terms of utilitarian responses to the IC dilemmas, but also dilemmas that we would argue show response patterns that undermine their validity.

### Concern 2: Justification for Scale Structure

Our second concern is that the internal structure of the moral PDP might not be justified by data. To our knowledge, few tests for the appropriateness of this structure have been done using psychometric methods.^3^ In summary, we have some concerns with how combining the C and IC dilemmas into the *U* and *D* scales is justified. These include insufficient appraisal of overall structure and item functioning.

The formulae used to compute the *U* and *D* scores from responses to C and IC dilemmas are:

$${}^{4}\,U=P\,\left(\mathrm{Unacceptable}|\mathrm{Congruent}\right)$$

(1) -P⁢(Unacceptable∣Incongruent)

(2) $$D=\frac{P\,\left(\mathrm{Unacceptable}|\mathrm{Incongruent}\right)}{\left(1-U\right)}$$

where the probabilities represent within-subject averages of the respective dilemma sets with responses with values 1 indicating unacceptance and 0 acceptance. Additionally, the traditional bipolar scores (*TS*) reflecting the standard scoring of Greene’s (2007) high conflict moral dilemmas can be computed as:

(3) Traditional⁢Scoring⁢(TS)=P⁢(Unacceptable∣Incongruent)

An implicit assumption in PDP formulae is that all the dilemmas are “worthy” of combining together: aggregating just any binary responses would not of course make any sense, so they need to exhibit certain statistical relationships. One potential pitfall is that if some items do not correlate well with the scale sum, that would mean that the implied continuity in the parameters would hardly be justified. The moral PDP also assumes that each dilemma functions equally well. Thus the scoring procedure always gives, e.g., the Car Accident and Time Machine dilemmas (Conway and Gawronski, 2013, Appendix A, pp. 231–233) equal weights, as alluded to above. Scale development for individual differences is typically a labor intensive effort to empirically examine these properties, which we have not seen published in the moral PDP context.

Another concern here is the calculation of the *D* parameter, which involves division by another variable, namely the complement of the *U* parameter. To our knowledge, this makes it hard to evaluate how well the data ‘justifies’ the model, at least by using more traditional psychometric methods such as factor analysis or Rasch models. Later, we show that it is possible to evaluate the properties of *U* using such methods by expressing its formula in a different, but equivalent way (see Appendix A for algebraic proof).

### Concern 3: Insufficient Evidence for Reliability

Our next concern is that due to its structure, the PDP model does not easily allow one to check for the internal consistency of items (an index of within-participant agreement for multiple items of the instrument indicating a signal-to-noise ratio). Unlike in, e.g., many personality measures, the items of the PDP are not simply averaged together to form an aggregate score. This is likely the reason we have been unable to find usual indices of reliability such as Cronbach’s alpha or McDonald’s omega (calculated from hierarchial factor analysis, and unlike alpha, does not assume that each item functions equally well; equals alpha when assumptions of alpha are not violated) reported in the PDP literature. This is problematic because agnosticism about reliability makes evaluating sample sizes in power calculations difficult (see Williams and Zimmerman, 1989), as well as interpreting effect sizes (Wilkinson and Task Force on Statistical Inference APA Board of Scientific Affairs, 1999). Large amounts of measurement error (low reliability) can both make it harder to find existing relationships (type 2 error) and lead to spurious findings (type 1 error), both contributing to non-replicability (Loken and Gelman, 2017).

Later, we calculate reliability coefficients for PDP parameters in two ways. First, the alternative formulation for *U* mentioned above enables the application of standard psychometric methods. Second, to estimate reliability for *D* as well, we use the split-half permutation method (see Parsons et al., 2019). We use these methods on our own empirical sample and find quite low reliabilities for both of the parameters.

### Concern 4: Validity Evidence

The reliability issue is concerning, because sufficient reliability is a necessary precondition for validity (Cook and Beckman, 2006). We are also concerned about some pieces of the presented validity evidence for the moral PDP. The two main pieces of construct validity evidence are the correlations between different PDP scores, and their relationships with other theoretically relevant constructs (Conway and Gawronski, 2013). In our analyses we examine the former, but discuss the external correlations later.

First, C&G (and later Friesdorf et al., 2015, in a meta-analysis of the moral PDP) argued that the correlations between PDP parameters and the traditional (bipolar) scores (*TS*) were evidence of the confound in traditional moral dilemmas. *U* had a strong negative relationship with *TS*, whereas *D* had a strong positive relationship with *TS*. C&G claimed that “this finding not only corroborates the validity of the two PD parameters; it also suggests that the traditional bipolar index indeed confounds two distinct processes […]” (p. 223). In other words, these correlations are taken as proof that two separate processes strongly drive *TS*. We will later show that these correlations could stem from the properties of the scoring method alone.

**FIGURE 1:**
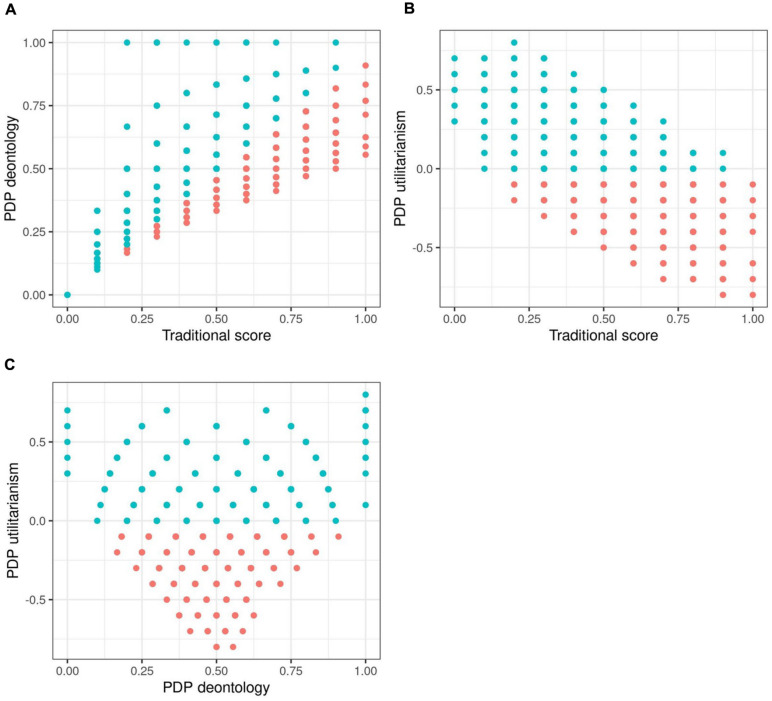
Scatterplots of process dissociation procedure (PDP) scores in randomly generated data absent of any real patterns. Blue dots represent simulated participants with realistic response distributions, with *U* parameter being > 0.

**FIGURE 2:**
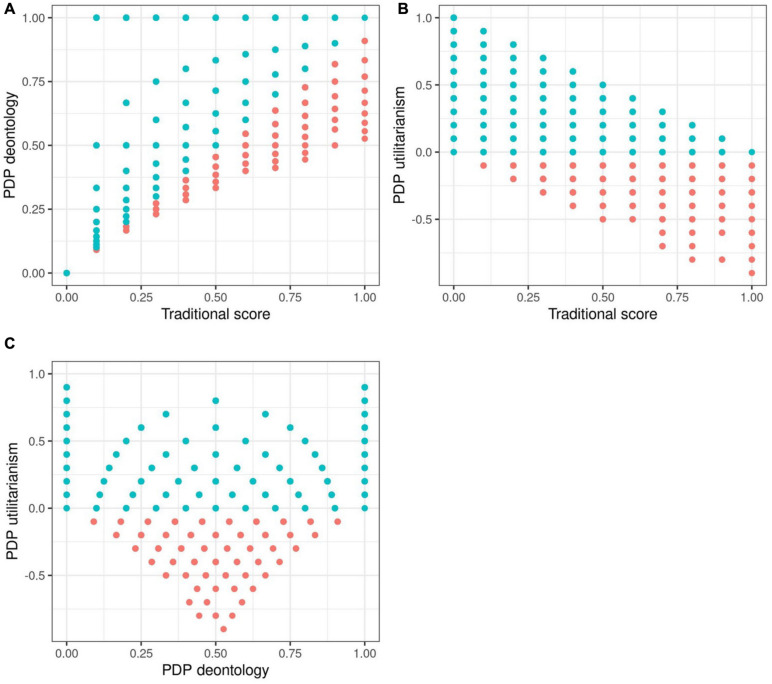
Scatterplots of process dissociation procedure (PDP) scores in simulation 2 simulated data with realistic assumptions. Blue dots represent simulated participants with realistic response distributions, with the *U* parameter being > 0.

**FIGURE 3:**
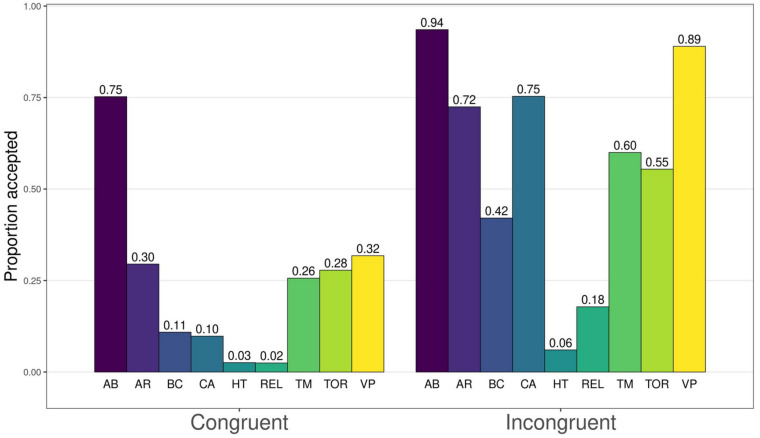
Proportions of accepted harm for congruent and incongruent dilemmas. AB, abortion; AR, animal research; BC, border crossing; CA, car accident; HT, hard times; REL, relationship; TM, time machine; TOR, torture; VP, vaccination policy.

In addition, C&G observed a nonexistent-to-small correlation between *U* and *D* parameters and considered this consistent with the separateness of two processes. However, we find this line of reasoning inaccurate because *U* is directly used in calculating *D* (see the formulas on the following section), and for this reason these cannot be considered truly independent, even in the absence of a (linear) correlation. This becomes apparent when one looks at the scatterplot between the *U* and *D* parameters (Figures 1C, 2C, 3C) where there is a clear non-random pattern stemming from the scoring formula. In our opinion, considering null correlation as evidence for separateness would require full distributional independence of the variables involved. We will elaborate on this later.

C&G presented several correlations to exogenous variables as evidence of construct validity. *D* had positive correlations with Empathetic Concern, Perspective-taking, Religiosity, and Moral Identity Internalization (MII), whereas *U* had a positive correlation with MII and a trending positive relationship with Need for Cognition. *TS* in turn had the same positive relationships as *D* with the exception of MII, and a negative trending relationship with MII. In addition, the authors showed that a cognitive load manipulation selectively influenced *U* but not *D*. These associations make sense in light of the dual-process model, but we have reservations about them based on our other concerns about how the PDP scoring works, which we cover in the discussion.

### Overview of the Data Analysis

Here we show, with two simulations and one empirical data set, that the PDP scoring method produces comparable results whether the data is purely random, simulated to be highly correlated and consistent with the theoretical assumption of lower acceptance of C dilemmas, or actual responses from real people. The results suggest that (1) some correlations between parameters are mathematical artifacts that emerge even from randomly generated data, (2) patterns are similar whether the data is randomly or “ideally” simulated, or actual responders, (3) reliabilities for all parameters calculated from PDP are poor, and (4) bipolar scores from PDP and a high-conflict moral dilemma battery (see Koenigs et al., 2007; Greene et al., 2008) might not reflect the same underlying construct. All the data, materials and scripts are available at https://osf.io/vmy4q/.

## Simulation 1

The purpose of SIMULATION 1 is to show that the correlations between the *TS* and *U* or *D* parameters of the moral PDP are mathematical artifacts. As stated earlier, C&G created moral PDP to be a “compelling test of the dominant dual-process account of moral judgment”, and the authors claimed these correlations confirm the existence of the confound between utilitarian and deontological processes. We show by simulation that these correlations would be very similar even when the data is completely random and there are no differences in response trends between the C and IC dilemmas. We argue that they cannot be considered as evidence for such claims.

### Methods

We simulated 10,000 responders with completely random response patterns with R (version 3.6.3) for the simulation. In other words, we simulate 10,000 rows of ten binary values with equal probabilities [meaning that *P*(*X* = 0) = 0.5 and *P*(*X* = 1) = 0.5] to represent responses for both C and IC dilemmas. A simulated response of 1 indicates that harm in the dilemma is considered unacceptable, and a response of 0 indicates that it is considered acceptable. The standard PDP scoring procedure is then used to compute *U, D*, and *TS* from this artificial data.

### Results

Correlations between the scores are presented in Table 1 and graphically in Figure 1, with blue dots indicating what we will term “realistic” responses: accepting the harm in IC dilemmas is more likely than in C dilemmas.^5^ We see that in completely random data, the *U* and *D* parameters exhibit substantial correlations with the unidimensional *TS*, in the directions observed by C&G and later ourselves in our empirical data. In Figure 1A, observations with high *TS* and low *D* parameters are absent below the diagonal and scarce on the upper left. This implies that the scoring does not allow any other results than a positive correlation between these two to emerge, provided the answering patterns have variance. Similarly, *TS* and *U* have a forced negative linear relationship, presented in Figure 1B.

**TABLE 1:** Correlations in completely randomly generated data with process dissociation procedure (PDP) scoring.

	***U***	***D***	***U***	***D***
*D*	0.00		0.02	
*TS*	−0.71***	0.68***	−0.53***	0.82***

D = PDP deontology parameter, U = PDP utilitarianism parameter, TS = PDP traditional bipolar utilitarianism-deontology score. The left-hand side of the table uses the whole simulated sample. The right-hand side uses only simulated responders with U greater than 0, a “realistic” response pattern (5831 out of 10000). ***p < 0.001.

We also observe the correlation between *U* and *D* parameters to be very close to zero. Note that the null correlation between *U* and *D* depends on variation in both IC and C dilemmas – of these, the variation in C dilemmas is the more theoretically interesting part. Variation in the IC dilemmas is to be expected as they are dilemmas where the two processes proposed by the dual process model drive different responses. In the C dilemmas, the processes are in agreement about a negative response. If responses to C dilemmas were a constant between participants, this would manifest as a strong negative correlation between *U* and *D* (see results from modified Simulation 1 in Appendix B). However, the significant correlations between *TS* and the two moral inclination parameters do not depend on variation in the C dilemmas: if *C* is set to be a constant, we would still observe a strong negative correlation between *U* and *TS* and a strong positive correlation between *D* and *TS*.

Since all parameters (including *TS*) are derived from the same sets of dilemmas, there are built-in dependencies between them. Thus, we argue that correlations between *TS* and the other parameters cannot be used as evidence for the separateness of the processes. These correlations would exist as long as there is variance in responses to IC dilemmas, and variance in IC dilemmas in and of itself is not evidence in favor of the dual-process model.

What is really notable in the scatterplots is the fan-shaped joint distribution of *D* and *U* (see Figure 1C). Although the correlation between *D* and *U* is zero, there is a clear non-random pattern between the parameters. This dependency is not surprising given *D* is calculated based on the value of *U*. We observe this also with empirical data. Even if there was no linear correlation between *U* and *D*, it is clear from the plot that if some manipulation would affect *U*, it would constrain or relax possible values for *D* as well. When *U* is at 0, *D* can have almost any value; when *U* increases from 0, the range of possible values for *D* narrow between the extremes. Extreme or midpoint *D* scores can co-occur with a wide range of *U* scores, but, e.g., a *D* score of 0.9 can only co-occur with a *U* score of 0. What follows from the relationship between *U* and *D* is that selectively increasing or decreasing *U* in a group of participants has an effect on the possible distribution of *D* scores. A lower value of *U* implies a wider range of possible values of *D*, despite theory stating the two should be independent. Affecting only *D* requires affecting C and IC dilemmas in the same direction and in the same degree; affecting only *U* is very difficult as *D* is dependent on both *U* and the absolute value of IC dilemmas (see the formulae presented in concern 2 and figures produced by simulation studies). Therefore, affecting a single parameter would require affecting both dilemma types in precisely the right way, and affecting a parameter truly selectively seems unlikely. This then means that it might be difficult to create manipulations that target only one process, and in many cases both are affected even if statistical significance is not achieved.^6^

To summarize, there are several features in the PDP that are artefactual in nature, which are either detrimental to theory-testing or theoretically implausible. These include correlations emerging from the scoring procedure, constraining patterns between different parameters, and a consequent difficulty in manipulating only one type of processing (as measured).

## Simulation 2

The purpose of Simulation 2 is to examine the same correlation patterns as before, but now in conditions where the data is very consistent. In brief, the results are basically the same as they were in Simulation 1 with purely random data. In Simulation 2, both C and IC dilemmas were simulated to have large correlations within their respective dilemma sets (but not between the dilemma sets). C dilemmas were set to be more unacceptable than IC dilemmas. See Table 2 and Figure 2 for the results.

**TABLE 2:** Correlations in simulated data with within-dilemma-set correlations.

	**U**	**D**	**U**	**D**
*D*	0.15***		0.11***	
*TS*	−0.66***	0.57***	−0.59***	0.66***

D = PDP deontology parameter; U = PDP utilitarianism parameter; TS = PDP traditional bipolar utilitarianism-deontology score; PDP = process dissociation procedure. The left-hand side of the table uses the whole simulated sample. The right-hand side uses only simulated responders with U greater than 0, a “realistic” response pattern (8,850 out of 10,000); *** = p < 0.001.

### Methods

Again, we simulated 10,000 responders with 20 binary values representing 10 C and 10 IC dilemmas. To ensure that these values would correlate similarly with one another, we initially simulated two sets of 10 correlated gaussian variables (mean = 0 and SD = 1; inter-item *r* = 0.50). We then separately dichotomized these to represent C and IC dilemma batteries by using different cut-off points for each dilemma resulting in different unacceptance rates. First, we randomly sampled the cut-off points for C dilemmas from uniform distribution (min = −1, max = 2). We then sampled the cut-off points for IC similarly, but made sure that the cut-off point for IC dilemma was always lower than for the corresponding C dilemma. We did this by lowering the minimum to −2, and using the previously sampled cut-off point for corresponding C dilemma as the maximum (min = −2, max = corresponding C cut-off point). This ensured that the probability of “unacceptable” responses was always greater for C dilemmas, and resulted in an overall unacceptance rate of 68% for C dilemmas, and 35% for IC dilemmas. We then applied PDP formulae to compute *U*, *D*, and *TS*.

### Results

The median correlation for dichotomized C dilemmas was 0.26 and for IC dilemmas 0.20. The correlations between the parameters are presented in Table 2, and corresponding scatterplots in Figure 2. The general pattern is the same: the *D* and *U* parameters correlate with *TS* in the theoretically predicted directions, but have a weak correlation with each other. It would thus seem that the issues we observed with random data are present with simulated consistent responses as well. In practical terms this implies, again, that the correlations between *TS* and the two parameters are caused by the scoring formulae, and specifically the inclusion of IC.

The range of possible *U* scores covers the whole spectrum of “realistic” values only when *D* is at either extreme. This pattern stems from the fact that the probability of accepting the harm in C and IC dilemmas constrains the possible values of the *U* and *D* parameters. Namely, 100% acceptance of the harm in IC dilemmas and 0% acceptance of the harm in C dilemmas are the only situations that allow for theoretically realistic responses so that the *U* parameter is not constrained. 100% acceptance in IC dilemmas corresponds to a *D* parameter value of 0, and 0% acceptance in C dilemmas corresponds a *D* parameter value of 1. We wish to emphasize that this constraining is a direct result of the way the PDP scoring works. This may be a lesser issue when using the PDP test for group-average contributions of two separate processes in a task as Jacoby (1991) did, but it leads to issues when using it as an individual differences measure. However, we also find the constraining generally problematic for the theory, given that it means that at face value, the processes as measured by the PDP do not and indeed cannot vary completely independently.

## Examination With Empirical Data

As established in Simulations 1 and 2, comparing PDP parameters with traditional bipolar scores when all are computed from PDP dilemmas presents a confound because the same items are used for all parameters. Here we examine a dataset collected for other purposes, which contains responses to both the PDP dilemmas and other high-conflict moral dilemmas (HCMDs; Greene et al., 2001; Koenigs et al., 2007). This enables us to examine correlations similar to those presented by C&G and our two simulations, but without the confound between *TS* and the *U* and *D* parameters because a different battery is used for *TS*. In addition, we use this data to examine the psychometric properties of the moral PDP. To summarize the results, we found low response consistencies in the PDP dilemma sets, which manifest as reliabilities below the accepted standards.

### Methods

#### Participants

A total of 1,043 participants were recruited from Prolific Academic^7^ to participate in an online experiment which is to be reported elsewhere. 33 participants were excluded from the sample due to failed attention checks. Of the retained sample, 466 were men, 535 women, 4 non-binary, and 5 refused to state their gender. Mean participant age was 37.35 (SD = 13.36).

#### Materials

##### PDP moral dilemmas

We presented participants with nine dilemma pairs from the moral PDP (Conway and Gawronski, 2013; we omitted the Crying Baby dilemma pair because a very similar dilemma was in the high-conflict dilemma battery). Each pair described similar situations with different consequences for an action. Participants were asked to indicate if they consider the action unacceptable. In C dilemmas, both consequences and norms encourage inaction; in IC dilemmas, norms encourage inaction while consequences encourage action. Traditional bipolar scores were computed by taking an average of the IC dilemmas. Higher traditional scores represent higher deontological responding and lower utilitarian responding. The *U* and *D* parameters were computed according to formulas from Conway and Gawronski (2013; based on Jacoby, 1991), presented in Simulation 1. Traditional scoring had a low reliability estimate (Cronbach’s alpha = 0.60, McDonald’s omega = 0.39; see below for *U* and *D* parameters; corresponding reliability estimates for only C dilemmas were: alpha = 0.71, and omega = 0.56).

##### High-conflict moral dilemmas

We presented participants with the 12 “high-conflict” (see Koenigs et al., 2007; Greene et al., 2008; see Laakasuo and Sundvall, 2016 for psychometric examination; see also Laakasuo et al., 2017) dilemmas from Greene et al.’s (2004) original dilemma battery: the traditional bipolar deontological/utilitarian scale. We asked participants to rate how acceptable the utilitarian solution to each dilemma was, on a scale from 1 (not at all acceptable) to 7 (totally acceptable), and averaged the responses to compute the bipolar score. Higher scores represent higher utilitarian and lower deontological tendencies (note that this scoring goes the opposite way compared to the PDP traditional score). The scale had a reliability estimate alpha of 0.88, and omega of 0.74.

### Results

#### Descriptives

On average, participants accepted the harm 56.8% of the time in IC dilemmas and 23.9% of the time in C dilemmas, broadly replicating C&G’s results. Proportions of responses where the participant accepted the harm in a PDP dilemma are presented in Figure 3 (see Conway and Gawronski, 2013, Appendix A, pp. 231–233, for the content of each dilemma pair). As expected, in all cases harm in C dilemmas was rated unacceptable more often than in IC dilemmas, though with considerable variation in both acceptance rates and the size of the difference within a dilemma pair. We also computed the proportions of participants who either changed or maintained their response (positive or negative) between the C and IC versions of each dilemma (Table 3). We did this to provide an overview of the “difficulty”^8^ of different dilemmas and potential problematic items. For each dilemma pair, only a small minority (under 5% of participants) gave responses where the C version was found acceptable but the IC version was not, i.e., an obviously problematic response.

**FIGURE 4:**
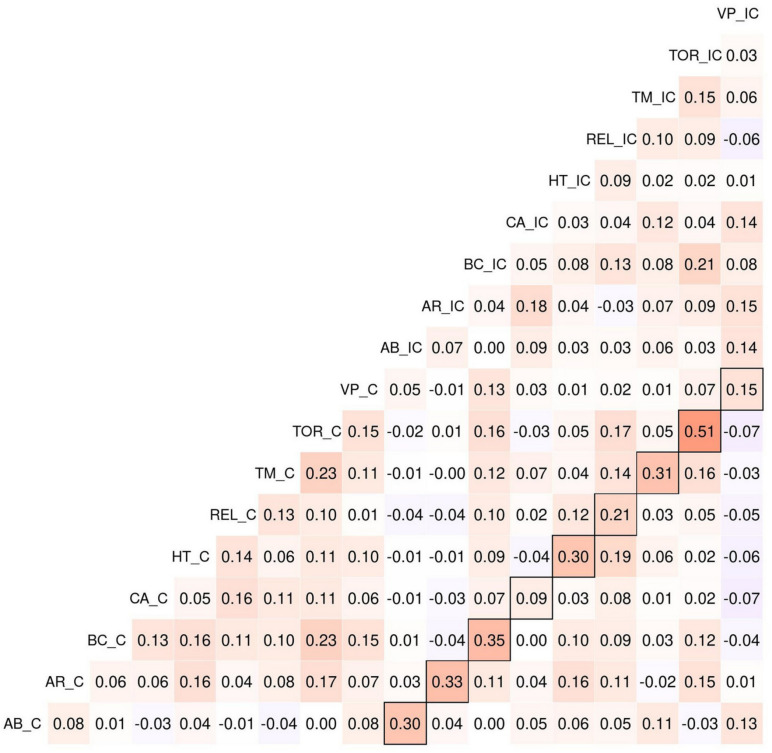
Spearman correlations between process dissociation procedure (PDP) dilemmas in empirical data. Contours indicate correlations for same dilemmas from C and IC dilemma sets. Note that the correlations for the large part are highest between the dilemma pairs and not within the incongruent (IC) and congruent (C) clusters.

**TABLE 3:** Proportions of different response patterns to congruent (C) and incongruent (IC) versions of dilemmas.

	**AB**	**AR**	**BC**	**CA**	**HT**	**REL**	**TM**	**TOR**	**VP**
Both unacceptable	0.048	0.260	0.569	0.233	0.928	0.814	0.363	0.436	0.097
Both acceptable	0.736	0.280	0.099	0.085	0.013	0.017	0.220	0.268	0.305
IC acceptable, C unacceptable	0.200	0.444	0.321	0.668	0.048	0.161	0.380	0.286	0.585
IC unacceptable, C acceptable	0.017	0.015	0.010	0.013	0.013	0.008	0.037	0.010	0.013
Difference	0.183	0.429	0.311	0.655	0.034	0.153	0.343	0.276	0.572

*AB = Abortion, AR = Animal research, BC = Border crossing, CA = Car accident, HT = Hard times, REL = Relationship, TM = Time machine, TOR = Torture, VP = Vaccination policy, Difference = P(accept—IC) - P(accept—C).*

In the Car Accident and Vaccine Policy dilemmas, over half of the participants responded “unacceptable” to the C version and “acceptable” to the IC version. Thus, in these dilemmas the congruency manipulation caused a difference for acceptability in a sensible direction for a majority of the sample, suggesting they were relatively “easy” in terms of utilitarianism (note that it is not necessary for a dilemma pair to have over 50% of participants responding in this way for the dilemma pair to be “good” or appropriate). A further two dilemmas, Animal Research and Time Machine, also had more responses in this category than any other category, but not over half the sample: the responses were more evenly spread between the other response patterns.

In the Border Crossing, Hard Times and Relationship dilemmas, over half of the participants responded “unacceptable” to both versions of the dilemma. In other words, a majority of the participants found the harm unacceptable in these dilemmas whether it had a utilitarian justification or not. Thus, these dilemmas seem to be the “hardest” in terms of utilitarianism. The Torture dilemma also had more “both unacceptable” responses than responses of any other type, but not over half the sample.

It is not necessarily clear why some dilemmas are “easy” or “hard.” A majority of “both unacceptable” responses may stem from the relevant deontological norm being very strong. However, it could also be that the utilitarian justification in some of the dilemmas was not very strong, or participants interpreted the dilemma differently from what the developers intended. For example, the utilitarian motivation in the Border Crossing IC scenario is only that a soldier has a *suspicion* that a person approaching a checkpoint intends to bomb the checkpoint and kill an unstated number of people: participants may not find this a clear enough motive to shoot the person. Similarly, a majority of “IC acceptable, C unacceptable” responses may stem from a strong utilitarian motive or a weak deontological norm.

The Abortion dilemma is an outlier, with a large majority (73.6%) of participants responding “acceptable” to both versions of the dilemma. This dilemma seems to be the only “hard” dilemma for rejecting harm in our sample. We suggest this stems either from a majority not actually recognizing a deontological norm against abortion, or from both versions of the dilemma having consequences that people may find worse than or equally as bad as abortion.^9^

In addition to the above, the Animal Research, Time Machine, Torture and Vaccine Policy dilemmas all had over 20% of “both acceptable” responses. For the Animal Research dilemma, we suggest that a norm against animal testing is not nearly as recognized as other deontological norms, and thus even the C version of the dilemma (animal testing for an acne medication) has notable acceptance. For the Vaccine Policy dilemma, we are less sure: accepting the C version of this dilemma means accepting a potentially lethal medicine to a non-lethal case of the flu. This may be a case of participants misinterpreting the dilemma in some way. The Time Machine and Torture dilemmas especially raise questions of validity, given that the C versions of these dilemmas deal with murder and torture, respectively. We argue that at face value, it is not plausible that over 25% of people have deontological and/or utilitarian inclinations that are too weak to condemn murder or torture without a utilitarian motivation. Rather, we think some participants in these two dilemmas may interpret the benefits of the harm (prevention of a child kidnapping for ransom or a bombing that vandalizes private property, respectively) as greater than intended by the developers of the measure.

In sum, in addition to the dilemmas having different levels of “difficulty” (Figure 3 and Table 3), there are several dilemmas where a large number of participants respond in a way that is hard to interpret. Differences in difficulty (inferred from response patterns, not self-reported by participants) are not inherently problematic, but experimenters should be aware of them, and consider what it means to create an average score out of different items without weighing the “easy” and “hard” items differently. At least some of the more problematic responses suggest that some participants may interpret dilemmas differently from what is intended, as suggested by Baron and Goodwin (2020). Given that a null correlation between the *U* and *D* parameters depends on variation between participants in responses to the C dilemmas, issues in interpretation pose a validity problem not only for specific dilemmas. The null correlation, which on its face supports the dual-process model, may arise from variation in C dilemmas that is not caused by moral inclinations but by differences in understanding the items.

#### Correlations Between PDP Dilemmas

We computed Spearman correlations between all PDP dilemmas to learn about the degree of response consistency (see Figure 4). First, in our sample, the median absolute correlation between dilemmas was 0.06, and the average absolute correlation was 0.08. This is concerning because co-variation among items is usually the basis for using them in a measurement scale.

**FIGURE 5:**
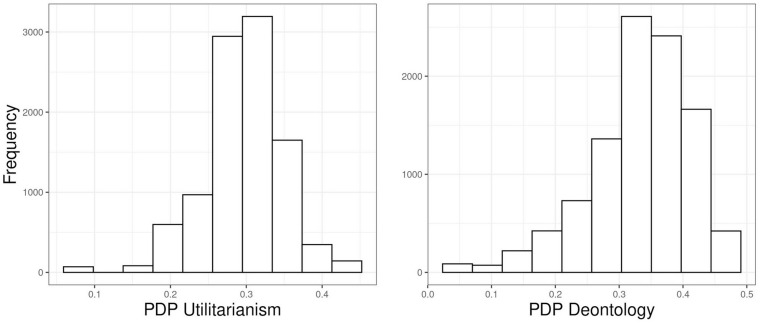
Distributions for reliability estimates for *U* and *D* from split-half permutations.

We also observe a similar non-linear relationship between *U* and *D* as with the “realistic” responses in Simulations 1 and 2. The “floor” of *U* was -0.2 in the empirical data (see Figure 5), due to a handful of participants (18 out of 1,010) who were more accepting in C than in IC dilemmas. The fact that our empirical data had very few “unrealistic” responses suggests that a majority of responders were generally logical in their responding, i.e., not accepting “useless” harms more than utilitarian ones (see Figure 4). Nevertheless, even with real responders, the distribution of *U* and *D* shows the theoretically implausible constraining between *U* and *D* seen in Simulations 1 and 2. As stated before, at face value, this is hard to reconcile with the claim of independent processes.

#### Reliability Analysis

Due to the nature of the PDP formulae, computing reliability coefficients (such as Cronbach’s alpha or McDonald’s omega) from the data is not as straightforward as in most questionnaires. The *U* scale can be composed as C dilemmas and reverse-coded IC dilemmas minus a constant (see Appendix A for mathematical proof). The constant in the formula should not affect statistical properties other than mean, so we can use this scale in psychometric analyses. This formulation has a Cronbach’s alpha of 0.16, and omega 0.08, that is, almost nonexistent (however, see permutation method below). However, if the IC dilemmas are not reversed (contrary to the PDP formula) this reliability coefficient becomes 0.77 (and omega 0.37). This may reflect deontological dispositions driving similar responses across the dilemma types, but either of these coefficients is sub-par. It is unclear what this formulation of the scale would represent: as stated, common psychometric assessments are hard to apply to the PDP.

Exploratory factor analysis (see Appendix C) for *U* suggests that almost all dilemmas load positively on a single factor (however, some with very low loadings) rather than IC dilemmas loading negatively. We also used confirmatory factor analysis with DWLS estimation for dichotomous items, but the pattern was essentially the same. If we were to interpret this as a kind of general moral condemnation factor – as deontology drives not accepting the harm in both dilemma types and utilitarianism in the C dilemmas – it is worrying that the loadings of the C dilemmas are not much better than those of the IC dilemmas.^10^

To gauge the reliability of the parameters we also used a permutation approach to split-half reliability for both *U* and *D* (as recommended by Parsons et al., 2019). This method investigates the response consistency of a scale by how similar responses are to the two halves of the same test. We randomly sampled 4 dilemma pairs and computed *U* and *D* parameters from these, and did the same for the remaining five dilemma pairs. We then computed the Spearman correlation coefficient between the parameters from the two halves. We iterated this process 10,000 times and then applied the Spearman–Brown correction (see Parsons et al., 2019) to these correlations to take into account underestimation of reliability.

**FIGURE 6:**
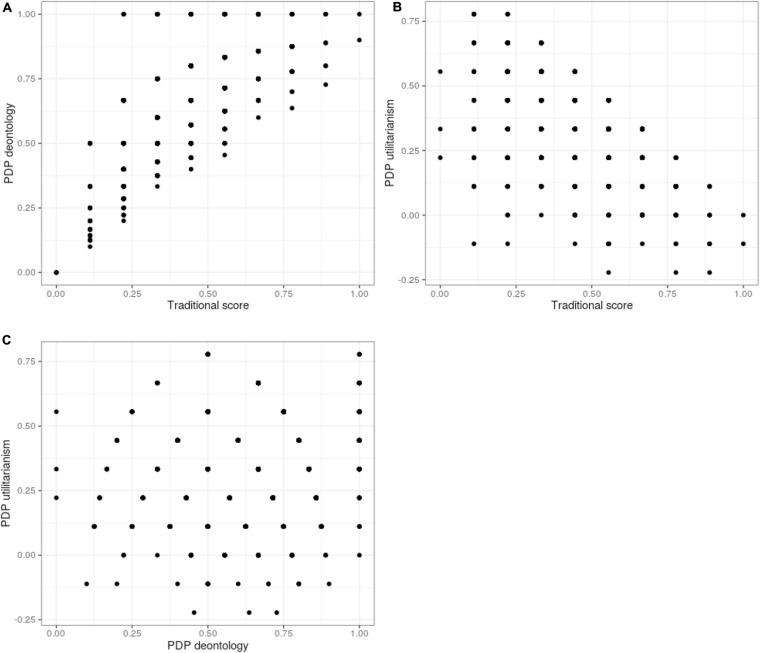
Scatterplots for process dissociation procedure (PDP) parameters from empirical data.

The resulting distribution of corrected estimates is presented in Figure 6. These function as direct estimates of reliability for these two scales, and should approximate Cronbach’s alpha (see Parsons et al., 2019). The average corrected relationship between the split-halves was 0.30 (SD = 0.05) for *U*, and 0.33 (SD = 0.07) for *D*. This reliability estimate for *U* does not converge well with coefficient alpha calculated above. Still, these results imply serious measurement inconsistencies for both *U* and *D*.^11^ For practical purposes, we believe these measures can hardly tap into any common cognitive, motivational or personality factors.

#### Comparison Between PDP and HCMD

The bipolar scores calculated from the two dilemma batteries (PDP and HCMD) should correlate very highly, as they are intended to be parallel measures of the exact same underlying trait. Additionally, *U* and *D* scores should exhibit similar correlation patterns with the HCMD scores as they do with *TS* computed from the PDP dilemmas.

The correlations are presented in Table 4 (for scatterplots between PDP scores, see Figure 6). We observe correlational patterns that are in the correct direction. These correlations are much lower than within PDP, but still non-trivial. A correlation of 0.51 between the HCMD *TS* and PDP *TS* means that only 26% of reliable variance is shared between the two measures. This implies heterogeneity in the measures that are supposed to reflect the exact same underlying trait, and thus undermining their equivalence. Some of this discrepancy is likely attributable to large measurement error in PDP scores. However, considering both the low reliability of PDP *TS* and this correlation, it seems that we cannot conclude that these two measures appropriately assess the same construct. If they do, and one of the measures has been incorrectly conceived in the past, it is not clear that the problem lies with the HCMD measure, which the moral PDP aimed to improve.

**TABLE 4:** Correlations between process dissociation procedure (PDP) parameters and high-conflict moral dilemma (HCMD) scores.

	***U***	***D***	**PDP TS**
*D*	0.12***		
PDP TS	−0.59***	0.70***	
HCMD TS	0.22***	−0.43***	−0.51***

D = PDP deontology parameter, U = PDP utilitarianism parameter, PDP TS = PDP traditional bipolar utilitarianism-deontology score, HCMD TS = HCMD bipolar utilitarianism-deontology score. Note that HCMD TS scoring is in reverse relative to PDP TS, thus a negative correlation between them is expected. ***p < 0.001.

### Summary

To summarize the results and implications of the analyses presented above, they indicate the following: first, there are severe response inconsistencies in the PDP dilemma sets that manifest as low correlations inconsistent in sign. Second, all parameters calculated from the PDP dilemma sets have sub-par reliability when examined with either coefficient alpha, coefficient omega, or the split-half permutation method. Third, there are nevertheless non-trivial correlations to the expected direction between the HCMD bipolar score and the PDP scores. Fourth, the correlation between the two different bipolar scores is still too low to ensure that the two measures tap into the same construct. Based on the results, we are skeptical whether the current formulation of PDP is able to quantify either individual utilitarianism and deontology or their bipolar continuum.

## Discussion

In this paper we have presented both theoretical and statistical concerns about the PDP measures of utilitarian and deontological tendencies, and using both simulations and empirical data, examined their psychometric properties. In Simulation 1, we showed that the PDP formulae produce similar correlations between the PDP parameters and the bipolar scoring method as observed in the empirical literature even when the data is randomly generated. In Simulation 2, we showed that the theoretically problematic non-independent distribution of data similar to Simulation 1 is also present when responses are highly reliable and aligned with theory. In our empirical data, we replicated the correlations between parameters found in prior literature and our simulations, and also found issues with reliability and similar distribution patterns as we did in our simulations.

Based on our results, the PDP scoring procedure constrains the possible values for the estimates of utilitarian and deontological inclinations. The scoring also leads to utilitarian and deontological scores that correlate with the traditional bipolar score. It is important to emphasize that these correlations are similar regardless of whether the data are completely random, simulated to be highly internally correlated and theoretically consistent, or actual responses from real people. They are also similar regardless of whether simulated “unrealistic” responses are excluded or not, and whether simulated participants vary in their responses to the congruent dilemmas or not. All this suggests that such correlations should not be interpreted as sufficient validity evidence for the scale, or an underlying theory. Moreover, in empirical data, the correlation between bipolar scores from the PDP and HCMD batteries is notable but low given that the scales should measure the same construct (*r* = 0.51). Correlations above 0.70 are recommended to claim that two instruments measure the same construct (Carlson and Herdman, 2012). This does not mean that the PDP necessarily measures something conceptually radically different from the HCMD battery: the low correlation may be caused by measurement error.

Our empirical data broadly replicates the results reported by C&G with harm in 57% of IC dilemmas 24% of C dilemmas being approved on average. The dilemmas are thus clearly not producing completely random responses from participants, and the difference between the two dilemma types makes theoretical sense. Another argument in favor of this is that in our empirical data we observed only few “unrealistic” responses, i.e., participants more approving of non-utilitarian than utilitarian harm. We do not wish to give the impression that the dilemmas are completely useless. The issue is that despite the theoretically expected difference between C and IC dilemmas, they produce, at least in our data, very noisy measurements of utilitarian or deontological tendencies within an individual. We argue this noisiness is not due to unmotivated or malignant responders, because as we mentioned previously, our sample had very few “unrealistic” responses. Averaging dilemmas that work very differently (e.g., due to tapping potentially mutually independent norms) produce noisy estimates of inclinations, which is the reason approaches like CFA and SEM are often used.

However, even with different weights on different dilemmas and/or elimination of unclear dilemmas, the PDP scoring itself causes issues. As mentioned above, we also replicated the correlation patterns between the *U*, *D* and *TS* parameters calculated from the PDP dilemmas in our empirical data. In light of the simulations and the examination of parameter reliabilities, our interpretation of these correlations between parameters calculated from PDP dilemmas is that they are mostly artifacts of the scoring procedure. We do not think the correlations between either of the two main parameters and the bipolar score are evidence in favor of the dual-process theory (contra C&G and Friesdorf et al., 2015). The null correlation between *U* and *D could* be used as evidence in favor of the dual-process theory, as it does not inevitably follow from the scoring. The lack of a correlation between *U* and *D* depends crucially on variation between individuals in responses to the C dilemmas, i.e., variation in what Baron and Goodwin (2020) term “perverse responses” (accepting harm that breaks norms and is not justified by its consequences). Our empirical data agrees with C&G and Friesdorf et al. (2015) in that people do, in fact, vary in their rates of “perverse responses”. If responses to the C dilemmas were constant between participants (but not with exactly 0% acceptance, as this would lead to no variance in the *D* parameter) in addition to variation in the IC dilemmas, the correlation between *U* and *D* would be strongly negative. Of course, if responses to the C dilemmas were a constant, there would be little reason to try and dissociate two processes in this way, as then the C dilemmas could simply be dropped from the procedure.

The theoretical issue here is that “perverse responses” may stem from several different factors, not all of them consistent with the theoretical reasoning behind the PDP or similar models, as Baron and Goodwin (2020) argued. That is, participants could have both utilitarian and deontological inclinations that are too weak to condemn the harm, or the norm against the harm could be quite weak – but participants could also simply disagree about the relevant norms, harms and consequences, or read carelessly. For example, does the relatively high acceptance of the congruent version of the Torture dilemma in our sample tell us about weak norms against torture, about disagreement about whether preventing vandalizing of private property is a good enough consequence to justify torture, or simply about misreading the dilemma? Additionally, at face value, it seems to us that there would be differences between some cultural or political groups, such as liberals and conservatives, on some of the dilemmas, regarding whether a norm against, e.g., animal testing or abortion actually exists, or how much of a harm these things are. For the instrument to work as intended, it should be measuring the relative contributions of, e.g., two cognitive processes or personality traits, not cultural effects. Note that this concern is separate from the wider discussion of whether utilitarianism or deontology measured using dilemma batteries map onto the philosophy of utilitarianism or deontology: our concern is measurement. We believe the HCMD dilemma battery side-steps this issue as a majority of the dilemmas are about causing the death of another human being in order to save others: the norm against killing is quite universal.

Due to the aforementioned issues, we would advise caution when interpreting the results of moral PDP studies. First, low reliabilities can increase risk for both spurious findings and non-findings (Loken and Gelman, 2017). Second, we argue that the constrained distribution patterns between parameters are enough to question results for individual parameters. If selectively affecting either parameter would constrain or relax values the other one could get, there are likely effects for the other parameter as well even if it does not reach statistical significance. Of course, the latter applies to measures derived from PD models more generally if similar scoring procedures are used to compute individual scores. We would advise caution in interpreting correlations between PDP parameters in these cases as their formulae can by themselves create artefactual associations.

Despite the PDP parameters having very low reliabilities, we observed them to have non-trivial correlations with the HCMD score, which can be interpreted in at least two ways. The first one is that the real correlations are large enough to remain notable even after dilution by measurement error. The other one is that there are limitations in our reliability estimation. The permutation method of split-half reliability is supposed to approximate Cronbach’s alpha, which acts as a lower-bound of true reliability. Observing very low reliability estimates thus leaves a wide range of possible values for true reliabilities. Thus, we consider the estimates provided here as the bare minimum as we found no other suitable methods for estimating the reliability of *D*.

We must note that there have been consistent results showing connections between specific moral PDP parameters and external variables such as gender (Friesdorf et al., 2015) or reasoning style (Conway and Gawronski, 2013; Byrd and Conway, 2019; Patil et al., 2020). As we mentioned earlier, an argument in favor of the moral PDP is that it seems to replicate results obtained with the more traditional bipolar measures of utilitarian judgment, and is related to variables measuring, e.g., reasoning in a way that makes sense in light of the dual-process model. However, there is some uncertainty about the dual-process model itself, which raises questions about the extent to which results that make sense in the light of that model support the PDP. Some recent publications question some of the bases of the dual-process model especially when it comes to emotions as a basis of moral judgment (e.g., McAuliffe, 2019; Rosas et al., 2019a,b). Regardless, we argue that at least some of the issues we have brought up here do not stand or fall based on how well a given result with the PDP replicates. Our concerns regarding the distributions and correlations between parameters that are forced by the scoring procedure are not invalidated by well-replicating results. Any relationship between the parameters and external variables does not take away the observation that the constraining between *U* and *D* is problematic.

Additionally, we argue that our concern about the reliability of the dilemma sets is not nullified by existing theoretically meaningful correlations between PDP measures and external variables, because the measure may still be noisy. As an analogous point, Gawronski et al. (2017) reported an effect of cognitive load on one of the CNI parameters, but as pointed out by Baron and Goodwin (2020), this seems to be driven by a single item. Given the variance in the PDP dilemmas, something similar may well happen there: individual differences driven mostly by one or two specific dilemmas. Ideally, effects should be assessed on the level of individual dilemmas or dilemma pairs (McGuire et al., 2009). As our empirical data shows (see Figure 4 for a plot of accepting harm in the C and IC dilemmas), there is great variance between the dilemmas, and it is not a *priori* clear why.

Moreover, even consistent associations between external variables and the whole set of PDP dilemmas may stem from these external factors affecting something else than moral reasoning per se. For example, there could be systematic differences in how carelessly participants respond to the dilemmas, or how often they disagree with the developers of the scale on what counts as a harm, a benefit, or a moral norm. This was pointed out by Baron and Goodwin (2020) in relation to the CNI model and sex differences, but the argument applies to the PDP and to other individual differences as well. For example, an association between the *U* parameter (but not the *D* parameter) and success in the cognitive reflection test (Patil et al., 2020) could be because people higher in reflection might read the congruent dilemmas more carefully and give fewer erroneous accepting responses as a result,^12^ instead of or in addition to other possible effects. As another example, the foreign language effect, where participants accept harm more often in congruent dilemmas not in their native tongue (see, e.g., Muda et al., 2018) would similarly make sense under the assumption that participants give more “perverse responses” when they misread a dilemma. Almost by definition, any individual difference involves fluctuations in the numbers of positive responses to the congruent dilemmas, which are hard to interpret as they may stem from several different factors. In short, while some effects may replicate well, it is not clear what is being replicated. We do not intend to claim that every well-replicating association between the PDP parameters and theoretically meaningful exogenous variables is an artifact. For example, we find it at face value believable that psychopathic traits are associated with less care for deontological norms (see, e.g., Reynolds and Conway, 2018). In any case, researchers should be careful to make sure that differences in a trait between groups stem from true differences in that trait and not a difference in how a measurement error in an instrument works for those groups.

More generally, while we found that mathematically speaking, things such as factor analysis can be applied to the *U* parameter of the moral PDP, it is not as clear that this makes sense from a substance perspective. That is, the scoring formula for the *U* parameter is equivalent to a sum score with reverse-coded IC dilemmas, which makes it possible to apply factor analysis. However, on the substance level, the items in the *U* parameter are each supposed to measure two separate latent variables, with a difference between the C and IC items in the way they load onto these variables. We are not currently aware of a method like factor analysis that would allow for confirming a structure like this. We have tried to provide a variety of approaches to assessing reliability, but due to the PDP approach being very different from measures psychometrics usually deals with, some of the analyses presented here may not be appropriate for assessing how well the utilitarianism/deontology PDP works. However, if this is the case, we are simply left in the dark: we do not even know how to assess whether moral dilemmas meant to measure utilitarian and deontological thinking do so in a consistent manner.

Notwithstanding the limitations, this paper has made novel methodological contributions in the psychometrics of utilitarianism and deontology. First, we have mathematically shown that the *U* parameter of the moral PDP can be evaluated using psychometric methods. Second, we have demonstrated that estimated measurement accuracy for both *U* and *D* can be assessed with the split-half permutation method. Moreover, we have shown that the PDP scoring formulae can produce very high artefactual correlations that can be misinterpreted as evidence for the dual-process theory.

## Conclusion

Conway and Gawronski (2013) raised an important methodological issue within moral psychology, which we believe is still very relevant. We have tried to elucidate in this paper why we think this issue cannot be solved with the PDP in its current form. We found several pieces of validity evidence either lacking or artefactual, or when investigated, insufficient. This suggests that either revisions to the model or novel methodologies are required to appropriately test for the existence of two separate processes and measure them.

## Data Availability Statement

The dataset, along with other materials, can be found online at https://osf.io/vmy4q/.

## Ethics Statement

The studies involving human participants were reviewed and approved by University of Helsinki Ethical Review Board in Humanities and Social and Behavioral Sciences. The patients/participants provided their written informed consent to participate in this study.

## Author Contributions

AK and ML conceived the initial study idea and initially analyzed the data. AK and JS drafted the first study and created simulations. ML collected the data. AK prepared the online materials. All authors contributed significantly to the improvement of the manuscript.

## Conflict of Interest

The authors declare that the research was conducted in the absence of any commercial or financial relationships that could be construed as a potential conflict of interest.

## Figures and Tables

**TABLE B1:** Correlations between process dissociation procedure (PDP) parameters when congruent (C) is held constant at 0.75 and incongruent (IC) is random.

	***U***	***D***	***U***	***D***
***D***	−0.94***		−0.95***	
**TS**	−1***	0.94***	−1***	0.95***

D = PDP deontology parameter, U = PDP utilitarianism parameter, TS = PDP traditional bipolar utilitarianism-deontology score. The left-hand side of the table uses the whole simulated sample. The right-hand side uses only simulated responders with U greater than 0, a “realistic” response pattern (9405 out of 10000). ***p < 0.001.

**TABLE:** 

**Dilemma**	**Loading**
Abortion (C)	0.06
Animal Research (C)	0.31
Border Crossing (C)	0.40
Car Accident (C)	0.21
Hard Times (C)	0.30
Relationship (C)	0.26
Time Machine (C)	0.37
Torture (C)	0.57
Vaccine Policy (C)	0.24
Abortion (IC)	0.05
Animal Research (IC)	0.10
Border Crossing (IC)	0.41
Car Accident (IC)	0.10
Hard Times (IC)	0.23
Relationship (IC)	0.33
Time Machine (IC)	0.22
Torture (IC)	0.47
Vaccine policy (IC)	0.01

*Factor analysis was conducted on tetrachoric correlation matrix due to variables being binary. No variables were reversed for this analysis. 9% variance was accounted for by the model; RMSR = 0.07.*

**TABLE:** 

**Dilemma**	**CFA Loading**
Abortion (C)	0.06
Animal research (C)	0.32
Border crossing (C)	0.35
Car accident (C)	0.18
Hard times (C)	0.19
Relationship (C)	0.18
Time machine (C)	0.38
Torture (C)	0.64
Vaccine policy (C)	0.21
Abortion (IC)	0.06
Animal research (IC)	0.17
Border crossing (IC)	0.39
Car accident (IC)	0.10
Hard times (IC)	0.14
Time machine (IC)	0.24
Torture (IC)	0.55
Relationship (IC)	0.28
Vaccine policy (IC)	0.03

*DWLS estimation (for dichotomous and categorical variables) was used in this confirmatory factor analysis (CFA). CFI: 0.65.*

## Footnotes

1 PD models have been popular with various dual-process theories (Sherman et al., 2014; Calanchini et al., 2018), and have been used for topics such as memory processes, judgment and decision making, object recognition, (implicit) social attitudes, and source monitoring (see Erdfelder et al., 2009; Payne and Bishara, 2009). However, it is important to note that simply applying these models do not by themselves reveal anything qualitative (like implicitness or explicitness) about these processes, and these claims must be established via manipulations and comparisons with external criteria (Sherman et al., 2014).

2 Robustness and replicability of experimental findings is facilitated by low between-subjects variability, but this variability is the very thing that measurement instruments attempt to capture. With low between-subjects variability there is very little to measure.

3 PD attempts to provide measures for any underlying processes in a way which in some ways resembles latent variable modeling: the task responses are used to infer the “hidden variables” that underlie the observed response patterns (Sherman et al., 2014). However, unlike more traditional psychometrics, PD models do not attempt to quantify the relationship of each task (or trial) with the unobserved process (like factor loadings or IRT difficulty parameters), and each will have equal weight in PD scores.

5 An important assumption of the PDP is that both of the two processes drive similar negative (i.e., not accepting harm) responses in the congruent condition, but different responses in the incongruent condition. A *U* score below 0 indicates that this assumption has been violated. Moreover, it indicates a participant who seemingly thinks, on average, that causing harm to others is *more acceptable when there is no aggregate benefit* than when there is such a benefit. We argue responses like this in real data would more likely indicate misunderstood task instructions or an unmotivated, malignant or a bot participant.

6 Note that this is an argument about mathematics, that is, about affecting a single process as measured by the instrument. However, there may also be a difficulty in truly affecting only one process conceptually, i.e., in the cognition of a participant, as there seem to be cases where, e.g., higher empathic concern predicts either more utilitarian or more deontological responding based on the type of moral dilemma (Rosas et al., 2019b).

7 http://www.prolific.co/

8 Note that we use the word “difficulty” here in a technical sense, referring to how common it is for participants to accept harm in the IC version and reject harm in the C version of a dilemma pair. This is different from subjectively assessed difficulty, i.e., how hard participants may have felt a dilemma was to answer.

9 In the incongruent version, the baby would die anyway if delivered. In the congruent version, it is stated that if the baby is delivered, the mother will spend the rest of her life as a single mother without a job, making life very hard for both her and the child.

10 Similarly to IQ test items, we should be capable of evaluating whether those cognitive tasks (here, moral judgments) load onto the same factor (i.e., that it can be claimed that they measure a similar construct).

11 In terms of signal-to-noise ratio, a reliability of 0.30 means there is 9% common variance among items, which means, there is 10 times as much noise as there is signal. As a reference, alpha of 0.71 would bring signal to noise ratio to about 1 (meaning that there is as much signal as there is noise).

12 Holding a hypothetical participant’s responses to IC dilemmas constant, and assuming only “realistic” responses, i.e., no negative *U* parameter, an increase in responding negatively to C dilemmas increases both the *U* and *D* parameters, but not to the same degree. The increase in *U* is always a linear increase of 0.1 for each additional negative response to a C dilemma (assuming 10 dilemma pairs), but the increase in *D* can be lower or higher than 0.1. Assuming a “realistic” response pattern, a move from the lowest “realistic” rate of negative responses to C dilemmas (given a specific rate of negative responses to IC dilemmas) to the highest will by definition increase *U* more than it does *D*, and could thus more likely lead to a significant effect on *U* but not *D*.

## Appendix A

### Algebraic Proof for the Alternative Expression for the U Formula

For any individual *i*, the formula can be expressed as:

$$1.U_{\text{i}}={\bar{x}}_{{\text{C}}_{\text{i}}}-{\bar{x}}_{{\text{IC}}_{\text{i}}}$$

Opening the formula for the latter average gives:

$$2.U_{i}={\bar{x}}_{C_{i}}-\frac{1}{k}\,\left(x_{{\text{IC}}_{i\,1}}+x_{{\text{IC}}_{i\,2}}+\ldots +x_{{\text{IC}}_{i\,k}}\right)$$

By reversing the sign in each IC dilemma response the formula becomes:

$$3.U_{i}={\bar{x}}_{C_{i}}+\frac{1}{k}\,\left(-x_{{\text{IC}}_{i\,1}}-x_{{\text{IC}}_{i\,2}}-\ldots -x_{{\text{IC}}_{i\,k}}\right)$$

By substituting −*x*IC*_i_**j* = −1 + (1−*x*IC*_i_**j*) for each incongruent dilemma *k:*

$$4.U_{i}={\bar{x}}_{C_{i}}+\frac{1}{k}\,\left(\left(-1+\left(1-x_{{\text{IC}}_{i\,1}}\right)\right)+\left(-1+\left(1-x_{{\text{IC}}_{i\,2}}\right)\right)+\ldots +\left(-1+\left(1-x_{{\text{IC}}_{i\,k}}\right)\right)\right)$$

Opening one layer of parentheses we get

$$5.U_{i}={\bar{x}}_{C_{i}}+\frac{1}{k}\,\left(-1+\left(1-x_{{\text{IC}}_{i\,1}}\right)-1+\left(1-x_{{\text{IC}}_{i\,2}}\right)+\ldots -1+\left(1-x_{{\text{IC}}_{i\,k}}\right)\right)$$

All -1 terms inside parentheses sum to -k:

$$6.U_{i}={\bar{x}}_{C_{i}}+\frac{1}{k}\,\left(-k+\left(1-x_{{\text{IC}}_{i\,1}}\right)+\left(1-x_{{\text{IC}}_{i\,2}}\right)+\ldots +\left(1-x_{{\text{IC}}_{i\,k}}\right)\right)$$

-*k* can be taken out of the parentheses, where it becomes -1 as it is multiplied by $\frac{1}{k}$:

$$7.U_{i}={\bar{x}}_{C_{i}}+\frac{1}{k}\,\left(\left(1-x_{{\text{IC}}_{i\,1}}\right)+\left(1-x_{{\text{IC}}_{i\,2}}\right)+\ldots +\left(1-x_{{\text{IC}}_{i\,k}}\right)\right)-1$$

Let’s notate *x*IC*_R_**_i_**_k_* = 1−*x*IC*_R_**_k_* as reverse-coded item *k* for person *i*

$$8.U_{i}={\bar{x}}_{C_{i}}+\frac{1}{k}\,\left(x_{{\text{IC}}_{R\,i\,1}}+x_{{\text{IC}}_{R\,i\,2}}+\ldots +x_{{\text{IC}}_{R\,i\,k}}\right)-1$$

Finally we can express the second term as an average again.

$$9.U_{i}={\bar{x}}_{C_{i}}+{\bar{x}}_{{\text{IC}}_{R\,i}}-1$$

Therefore *U* is the sum of average congruent response and average reverse-coded incongruent response minus a constant.
